# Genetic Testing for Antipsychotic Pharmacotherapy: Bench to Bedside

**DOI:** 10.3390/bs11070097

**Published:** 2021-06-30

**Authors:** Mujeeb U. Shad

**Affiliations:** 1 Spring Valley Hospital and Medical Center, Valley Health System, Las Vegas, NV 89118, USA; mujeebushad@gmail.com; 2 Department of Psychiatry, University of Nevada, Las Vegas, NV 89154, USA; 3 College of Osteopathic Medicine, Touro University Nevada, Las Vegas, NV 89014, USA

**Keywords:** genetic testing, antipsychotic, pharmacotherapy, schizophrenia

## Abstract

There is growing research interest in learning the genetic basis of response and adverse effects with psychotropic medications, including antipsychotic drugs. However, the clinical utility of information from genetic studies is compromised by their controversial results, primarily due to relatively small effect and sample sizes. Clinical, demographic, and environmental differences in patient cohorts further explain the lack of consistent results from these genetic studies. Furthermore, the availability of psychopharmacological expertise in interpreting clinically meaningful results from genetic assays has been a challenge, one that often results in suboptimal use of genetic testing in clinical practice. These limitations explain the difficulties in the translation of psychopharmacological research in pharmacogenetics and pharmacogenomics from bench to bedside to manage increasingly treatment-refractory psychiatric disorders, especially schizophrenia. Although these shortcomings question the utility of genetic testing in the general population, the commercially available genetic assays are being increasingly utilized to optimize the effectiveness of psychotropic medications in the treatment-refractory patient population, including schizophrenia. In this context, patients with treatment-refractory schizophrenia are among of the most vulnerable patients to be exposed to the debilitating adverse effects from often irrational and high-dose antipsychotic polypharmacy without clinically meaningful benefits. The primary objective of this comprehensive review is to analyze and interpret replicated findings from the genetic studies to identify specific genetic biomarkers that could be utilized to enhance antipsychotic efficacy and tolerability in the treatment-refractory schizophrenia population.

## 1. Introduction

Genetic testing is increasingly utilized to identify genetic biomarkers for optimizing the efficacy and tolerability of psychotropic drugs, especially antidepressants. However, genetic testing is also being requested to enhance the effectiveness of antipsychotic drugs, which is especially true for the treatment-refractory schizophrenia population, who frequently experience irrational polypharmacy at high dosages with significant adverse effects, generally without much therapeutic benefit. Currently, the most evidence in supporting genetic assays is based on pharmacogenetic (PG) studies, which utilize a candidate gene(s) approach investigating the effect(s) of genetic polymorphism(s) on efficacy, tolerability, and/or safety of antipsychotic drugs [1]. Most clinically meaningful findings have been reported using genetic factors affecting the pharmacokinetics (PKs) of antipsychotic drugs, such as genetic polymorphisms in the drug-metabolizing cytochrome-P450 (CYP) enzymes to identify and/or predict effective and tolerable dosages of an antipsychotic drug. In contrast, findings from PG studies investigating genetic variance in pharmacodynamic (PD) factors, such as transporters, neuropeptides, and receptors, have produced inconsistent results, which is most likely due to the small sample sizes (limiting sub-group analyses) resulting in small effect sizes, lack of control for confounding factors (affecting efficacy and/or tolerability), and medication nonadherence further compromising the results. Despite these limitations, PG studies investigating PD genetic factors may be helpful to enhance the effectiveness of antipsychotic drugs with more success in the treatment-refractory population. Compared to PG studies, pharmacogenomic (PGx) studies require much larger samples to explore all potential associations between the effectiveness of antipsychotic drugs and multiple genes of the molecular targets for the antipsychotic drugs. Although Genome-Wide Association Studies (GWAS) provide the first logical step to explore these associations, these studies can be significantly expensive, which explains their scarcity in psychiatry. The lack of optimal research explains difficulties in the clinical application of genetic findings from the bench to the bedside. This review is a comprehensive analysis of findings from a large number of positive and negative genetic studies to provide insights into specific genetic biomarkers that could be utilized to optimize antipsychotic efficacy and tolerability, especially in the treatment-refractory schizophrenia population. The review also offers a brief discussion of commercially available genetic assays and future research directions to enhance the clinical utility of genetic testing in psychiatry.

## 2. Pharmacogenetic Studies

The data from PG studies are clinically utilized at the individual level to predict and optimize the response to antipsychotic drugs while preventing or minimizing adverse events. A drug’s response or tolerability can be affected by genetic polymorphisms in PK factors, which determine the concentration of a drug at its site(s) of action, and PD factors, which determine a drug’s response or tolerability at its molecular targets. However, these distinctions are rather arbitrary, as changes in a drug’s concentration at the site of action (i.e., PKs) are always associated with changes in a drug’s efficacy and/or tolerability (i.e., PDs) at its site(s) of action. The following section will review the PK and PD genetic findings from the pharmacogenetic studies, followed by a brief discussion of pharmacogenomic studies, commercially available assays, and future directions.

### 2.1. Pharmacokinetic (PK) Genetic Biomarkers

Genetic variance in drug-metabolizing enzymes, such as CYP enzymes, represents most of the PK biomarkers. The genetic polymorphisms of CYP enzymes have produced one of the most replicated and clinically relevant findings in patients who develop adverse effects on routinely administered dosages of an antipsychotic drug. A similar statement cannot be made for antipsychotic efficacy, probably because there is no apparent relationship between plasma levels of an antipsychotic drug and antipsychotic response with the exception of clozapine. In this context, CYP2D6 is one of the most clinically relevant enzymes; despite making only 2% of all CYP enzymes in the liver, CYP2D6 is involved in the metabolism of about 25% of several commonly used psychotropic agents, including antipsychotic drugs [2,3]. About 6–10% of Caucasians and 1% of Asians are poor metabolizers [4]. Patients homozygous for wild-type alleles are known as normal or extensive metabolizers, and those homozygous or heterozygous for the dysfunctional allele are labeled as intermediate metabolizers. About 1–2% of Caucasians have multiple copies of functional alleles and are called ultra-rapid metabolizers [5,6]. As compared to extensive metabolizers, patients that are ultra-rapid metabolizers require higher doses and those who are intermediate metabolizers require lower doses of drugs that are substrates for this enzyme due to altered elimination. If antipsychotic doses are not corrected for this genetic variance, ultra-rapid metabolizers for CYP2D6 may experience decrease or loss in efficacy and poor metabolizers may develop higher levels of antipsychotic drugs resulting in adverse effects, such as extrapyramidal symptoms (EPS) and hyperprolactinemia [2]. Despite relatively small sample PG studies, multiple studies have shown a relationship between dysfunctional CYP2D6 variants and antipsychotic-induced EPS, especially tardive dyskinesia (TD) [7,8,9,10,11,12,13,14,15,16,17,18,19,20,21] (Table 1). However, these findings have not been supported in some ethnic groups, such as in Indian [22], Slovenian [23], and Japanese [24] populations. These differences may be explained by small sample sizes and a lower frequency of poor metabolizer alleles for CYP2D6 alleles in these ethnic groups as compared to Caucasians. Nevertheless, a meta-analysis revealed at least one dysfunctional CYP2D6 allele associated with TD and parkinsonian symptoms in patients with schizophrenia [25]. Interestingly, most of these PG studies reporting an association between antipsychotic drugs and EPS failed to show a significant correlation between CYP2D6 variants and the efficacy of antipsychotic drugs [3]. However, many studies have been small, and many have not been adequately powered to capture more subtle changes in efficacy compared to more clinically visible EPS.

Deficient activity of CYP enzyme *1A2* has also been associated with adverse effects due to an increase in plasma levels of antipsychotic drugs that are substrates for this enzyme, such as clozapine and olanzapine [21,42,43]. In contrast, patients with high inducibility of CYP1A2, as observed with smoking in some patients, may end up with subtherapeutic levels of clozapine and olanzapine [44]. One study associated genetic variance in CYP3A4 activity with the efficacy of risperidone, an antipsychotic drug [45], while other studies produced negative results [19,22]. However, polymorphism in a specific transporter, P-glycoprotein (also known as multiple drug resistance-1 (MDR1) or ATP-binding cassette subfamily B member1 gene [46]) has been correlated with efficacy as well as tolerability of risperidone [47] and clozapine [48].

### 2.2. Pharmacodynamic (PD) Biomarkers

#### 2.2.1. Antipsychotic Response

Antipsychotic efficacy across different antipsychotic drugs has been strongly linked with genetic variance in dopamine-2 receptors (DRD2). More specifically, *D2*−141C Del and TaqI A2 allelic variants have been associated with the inadequate antipsychotic response across various ethnic groups [49,50,51,52]. A comprehensive metanalysis supported the relationship between *D2*−141C Del and TaqI A2 allelic variants and antipsychotic response [26] (Table 1). Polymorphisms of the promotor regions of DRD2, DRD3, and DRD4 have also been linked with antipsychotic efficacy [52,53,54,55,56]. Another biomarker repeatedly associated with antipsychotic efficacy is catechol-o-methyl transferase (COMT), which primarily metabolizes dopamine [57,58,59,60] (Table 1). This finding was also supported by a meta-analysis [32], which showed that patients with met/met homozygosity were more likely to respond to antipsychotic drugs, especially the newer ones.

Specific polymorphisms in serotonin receptors have also been linked with the antipsychotic response, especially serotonin-*2A* receptors (HRT2A) 102-C/C genotype, which was associated with reduced antipsychotic efficacy in Caucasian patients [61,62] (Table 1). Another HTR2A genotype, 1438-A/A, has been correlated with antipsychotic response in various ethnic groups. Lack of antipsychotic efficacy and treatment resistance for negative symptoms were found in a French cohort with 5*-HT2A* −1438-A/A genotype [63]. In Algerian patients, *5-HT2A* −1438-G allele was associated with psychotic relapse [64]. Another polymorphism in serotonin receptor, HTR1A (i.e., *5-HT1A* −1019G), has been associated with lower antipsychotic efficacy in various ethnic groups [28,29,30]. The association between symptom reduction and guanine nucleotide-binding protein subunit beta-3 variant was reported in more than one study [65,66]. However, the correlation between the L allele of *5-HTT* LPR (serotonin transporter-linked promoter region) and improvement in negative symptoms of schizophrenia was observed in one study [67], while two other studies were negative [68,69]. Although multiple other reports have also observed association between specific PD markers and antipsychotic efficacy, these findings are without replication and questionable clinical utility [70,71,72,73,74,75,76,77,78,79].

Some studies have examined the pharmacogenetics of commonly used antipsychotic drugs, such as clozapine, risperidone, and olanzapine. In this context, clozapine, which is still the gold standard in the management of treatment-refractory schizophrenia, is the most extensively studied antipsychotic drug. Several studies have examined dopamine receptor polymorphisms to explain clozapine’s unique efficacy and have found replicated genetic variance in DRD1 [80,81], DRD2 [82,83], DRD3 [84,85], and DRD4 [86,87] to be associated with clozapine efficacy. However, results with DRD3 were not supported by a recent meta-analysis [88], while the findings with DRD4 were not replicated in other studies [89,90]. Association between clozapine’s efficacy and genetic variance in the dopamine transporter protein (DAT) has been supported by one study [91] but not the other [55]. However, one of the most robust findings with clozapine has been the correlation between HTR2A polymorphisms and clozapine treatment outcomes [62,92,93,94,95]. The results with HTR2A variants 102-T/C and Tyr452 were also confirmed by a meta-analysis [31] (Table 1). A comprehensive review documented correlations between antipsychotic efficacy and lower expression of HTR2A variants 102-C and -1438-G, and decreased functioning of HTR2A variant Tyr452 [1]. Other serotonin mechanisms have also been linked with clozapine’s efficacy, such as variance in serotonin-2C (HTR2C) receptors [96,97] and SLC6A4 (solute carrier family 6 member 4 serotonin transporter) [67,98]. A combinatorial genetic assay for three HTR2A variants (i.e., 452Tyr, 1438–G/A, and 102–T/C), two HTR2C variants (i.e., 330–GT/244–CT and Cys23Ser), and one SLC6A4 variant provides the best predictive model for clozapine response with 76.7% positive predictive value and 95% sensitivity [97,98]. Despite several studies producing negative results with polymorphisms in various serotonin targets [67,99,100,101,102,103,104,105,106,107], the overall data support the critical role of the serotonin system in clozapine’s efficacy. However, clozapine response has not been associated with genetic variance in other important clozapine-targeted receptors, such as adrenergic and glutamatergic receptors [108,109,110].

Risperidone is another second-generation antipsychotic drug, which has shown decreased antipsychotic efficacy in patients with DRD2 Ser311 [111] variant associated with the reduced response at DRD2 receptors [112]. On the other hand, the D2-241-A allele was associated with a greater antipsychotic response with risperidone than the −241-G allele in two studies [113,114]. A catechol-o-methyl transferase (*COMT)* variant (i.e., Val 158Met) has also been associated with lower risperidone efficacy in two studies [115,116] and a metanalysis [32] (Table 1) as has been associated with a serotonin receptor variant, HRT2A 102-C, in Chinese [117], Korean [118], and Japanese [119] patients, but not in Caucasians [1]. Nevertheless, this relationship between COMT variant and antipsychotic efficacy points towards the importance of dopamine levels in antipsychotic response. Risperidone efficacy has also been linked with variance in brain-derived neurotrophic factor (*BDNF*) in two studies [120,121]. However, unlike clozapine, no correlation was reported between risperidone response and *DRD4* variance [122]. Other genetic findings with risperidone have been in single studies and will not be reviewed here [28,53,113,119,123,124,125,126,127,128,129]. The findings from these single studies need to be replicated to be clinically relevant.

Olanzapine is another commonly used second-generation antipsychotic drug with reports of an association between its efficacy and DRD3 variant *D3*Ser9Gly [130,131], which has also been associated with antipsychotic efficacy of risperidone and clozapine [130,131]. However, this finding was not replicated in Indian patients [132], suggesting ethnic differences in response. Unlike risperidone, genetic variance in COMT was associated with olanzapine’s efficacy in only one study [133]. In terms of serotonergic mechanisms, none of the findings with variance in the L allele of the *5-HTT* LPR [134] and HTR6 polymorphisms [135] associated with olanzapine’s efficacy have been replicated. However, once again, this olanzapine response was not associated with HRT2A and HRT2C variants in the Indian population [131,132], highlighting the ethnic differences in antipsychotic response. Glutamate metabotropic receptor-3 polymorphism [136] associated with better olanzapine response in only one study, a positive olanzapine’s response with calcium channel variant, calcium voltage-gated channel subunit alpha1 C, rs1006737 was replicated in two studies [137,138].

Although aripiprazole is classified as one of the newer second-generation antipsychotic drugs, it is the first antipsychotic drug with partial agonist activity at D2 receptors and 5HT1A receptors [139]. A couple of studies have documented an association between *D2* TaqI variants and the efficacy of aripiprazole in Korean and Chinese patients [140,141]. In summary, there is inadequate genetic data to compare clinically meaningful differences in genetically mediated antipsychotic response between different antipsychotic drugs, perhaps with the exception of clozapine.

#### 2.2.2. Antipsychotic Adverse Effects

The genetic data for antipsychotic tolerability is not as consistent as those for antipsychotic efficacy, except for weight gain. The margin for controversial results is much higher than those from the efficacy studies, as documented below.

#### Extrapyramidal Symptoms (EPS)

Although genetic polymorphisms in CYP enzymes are grouped under PK biomarkers, it is worth mentioning here that any change in a drug’s metabolism will eventually be expressed pharmacodynamically. Thus, the poor metabolizers for CYP2D6 have a higher risk for developing EPS due to increased plasma levels of antipsychotic drugs that are CYP2D6 substrates [7,8,9,10,11,12,13,14,15,16,17,18,19,20,21] (Table 1). However, the relationship between D2Rs polymorphisms and the development of EPS remains unclear [40]. Although some studies have found a correlation between DRD2 variants and EPS [142,143,144,145], many others have not [19,146,147,148,149,150,151,152,153,154,155]. Nevertheless, a metanalysis did report a significant correlation between DRD2 polymorphism (i.e., TAq1A) and TD [40] (Table 1). The results examining relationship between EPS and DRD3 polymorphisms are also controversial; some studies supported the relationship [19,147,150,156,157,158,159,160,161,162,163], but some did not [143,147,150,164,165,166,167], while some strangely reported paradoxical results [168,169,170]. One study found an interaction between *DRD3* and CYP 17A1 genotypes and EPS [158]. Another study reported no correlation between variance in DRD1 and EPS [155]. A couple of studies found a direct association between two variants of dopamine metabolizing enzyme, COMT (G158A and A-278G) and risk for TD [148,171]. However, results were negative with another COMT variant, Val158Met [146,172,173,174]. No associations were reported with genetic variance in other dopamine targets, such as dopamine transporter-1 (DAT1) [146,147,175] and polymorphisms of dopamine-related enzymes, monoamine oxidase A, and monoamine oxidase B [146,174]. The relationship between the regulator of the G-protein signaling 2 gene and pseudo-parkinsonian symptoms was supported in Caucasian [176,177] as well as in Japanese [178] patients.

Genetic variance in the serotonergic system has also produced inconsistent results; some reports have documented associations between HRT2A polymorphisms and TD [150,170,179,180], and some have not [143,172,181,182]. However, pooled data from 635 patients reported a correlation between the HRT2A 102-C allele and age-related increase in risk for TD [39] (Table 1). A link between TD and HRT2C variant Cys23Ser was supported by several studies [143,163,183,184,185], but not all [109,143,163,181]. In addition, no relationship was discovered between EPS and HRT2A or serotonin transporter (SLC6A4) gene variants [172,186,187]. Although three studies linked polymorphism in the brain-derived neurotrophic factor (BDNF) gene with the risk of TD [120,165,188], one study produced negative results [168]. Polymorphism in a p-glycoprotein transporter gene, ATP-binding cassette sub-family B member 1 (ABCB1), was only marginally associated with dystonia and akathisia [189]. No clear associations were observed between EPS and genes involved in oxidation and stress, such as manganese superoxide dismutase [190,191,192], nitric oxide synthase [193,194,195], glutathione S-transferase [196], and glutathione peroxidase [197]. Only marginal associations were reported with polymorphism in nicotinamide adenine dinucleotide phosphate (NADPH), dehydrogenase quinone, nitric oxide synthase 3 [198,199], and glutathione S-transferase μ1 [19]. Polymorphisms in angiotensin I converting enzyme [156] and protein kinase B [200] were not found to correlate with EPS as well. However, in one study, EPS were associated with polymorphism in adrenergic type 1A receptors [201]. Despite inconsistent results from reviewed studies, the overall data do support an important role for dopamine and serotonin systems in the development of antipsychotic drugs-induced EPS.

#### Hyperprolactinemia

Although there is not much research investigating the role of genetic variance on antipsychotic-induced hyperprolactinemia, any DRD2 polymorphism that increases the risk for EPS will also increase the risk for hyperprolactinemia, as both adverse effects are mediated by D2R blockade. In this context, one study did report 40% higher prolactin levels in patients with DRD2*A1 allele than those without [202]. Interestingly, this increase in prolactin was also observed with clozapine, which is least likely to increase prolactin levels [202].

#### Weight Gain and Metabolic Syndrome

Serotonin is one of the main neurotransmitter systems that control feeding behavior in the hypothalamus and is targeted by the second-generation antipsychotic drugs via their blocking effects on 5HT2C receptors [203]. Although the genetic mechanisms underlying weight gain due to HTR2C polymorphisms are not completely clear, several HRT2C gene haplotypes have been associated with weight gain and metabolic syndrome [34,35,36,37]. Haplotype A (-997G, -759C, -697G) was the most robustly associated haplotype with antipsychotic-induced weight gain. In contrast, presence of haplotype B (-997A, -759T, -697C) was found to be protective against antipsychotic-induced weight gain [204,205,206,207,208,209,210] (Table 1). One of these haplotypes, B (i.e., -759T), has been associated with decreased expression of 5HT2C receptors [33]. Increased negative feedback due to increased levels of leptin observed in patients with haplotype B may explain the resistance against weight gain [27]. Interestingly this weight resistance with haplotype B was not observed in younger patients [211]. Although a meta-analysis revealed a 100% increase in risk for weight gain in patients with HRT2C -759 C allele [212], there were studies that did not find any correlation between the presence of the -759 C allele and weight gain [213,214,215,216,217]. In addition, no relationship was observed between weight gain and another HRT2C polymorphism, Cys23Ser [212,218,219]. Genetic variance in other serotonin receptors, such as HRT2A 102-T/C, have also been associated with weight gain, obesity, and lipid levels [34,220,221], except one study [219]. No association was documented with a HRT1A polymorphism [220]. Finally, a short allele of the serotonin transporter gene (i.e., *5-*H*TT* LPR) was significantly associated with weight gain and obesity in the Caucasian population [134,221], but not in Chinese patients [219].

Although earlier studies did find an association between weight gain and genetic variance in DRD2 [220,222] or DRD3 [220], one recent study did observe a positive relationship between weight gain and DRD2 variants rs6277 (C957T), rs1079598, and rs1800497 (TaqIA) [223]. In addition, a functional promoter region variant in DRD2 was implicated in a study of antipsychotic drug-induced weight gain during early psychosis with minimal prior exposure to antipsychotic drugs [224]. Carriers of –141C Ins/Del in the DRD2 promoter gene demonstrated substantially more weight gain than noncarriers after 6 weeks of treatment with risperidone or olanzapine. Another study reported an association between an increase in body mass index and a DRD4 variable number tandem repeat allele during antipsychotic treatment [218].

Few studies have reported a significant correlation between genetic polymorphism in melanocortin 4 receptors (MC4R) and antipsychotic-induced weight gain [225,226], which is also supported by a genome-wide association study [38] (Table 1). Several studies have reported involvement of the adrenergic receptor 2A in treatment-related weight gain, although with differential effects across various ethnic groups [227,228,229]. Genetic variance in other adrenergic receptors, such as 5HT1A, have also been associated with changes in body mass index [230]. Leptin appears to play an important role in mediating antipsychotic drug-induced weight gain, as reflected by the association between a leptin gene variant, −2548-A/G, and weight gain, despite the different direction of these results [209,216,231,232,233]. Results with leptin studies were also inconclusive across various ethnic groups, such as Indians [171] and Germans [37]. Interestingly, this leptin variant was not associated with weight gain in patients with premorbid obesity [234]. One study also found a correlation between a leptin receptor polymorphism and weight gain [234].

One study reported a correlation between antipsychotic-induced weight gain and polymorphism in insulin-induced gene 2 [235], but a couple of other studies did not [37,236]. Similarly, the association between guanine nucleotide-binding protein subunit beta-3 polymorphism and weight gain in Indians [132] was not replicated in other ethnic groups, such as Koreans [237], Taiwanese [238], and Caucasians [239]. One study failed to find any association between the histamine-1 receptor gene and antipsychotic-induced weight gain [240]. Results were also negative with the cholecystokinin gene [241]. However, associations have been reported between weight gain and/or metabolic syndrome and apolipoprotein E [242], brain-derived neurotrophic factor [220,243], cannabinoid receptor-1 [244], *CYP2D6* [220,245], multidrug resistance 1 [217], methylenetetrahydrofolate reductase [246,247], peroxisome proliferator-activated receptor-γ [248], synaptosomal-associated protein *25* [249], and tumor necrosis factor [250,251].

#### Agranulocytosis

Agranulocytosis is a rare but severe and potentially lethal adverse effect associated with clozapine use. Pharmacogenetic studies have reported strong associations between polymorphisms in the major histocompatibility complex and clozapine-induced agranulocytosis [252,253,254]. Two cohorts from a clozapine study found significantly high odds ratios (16.9) for agranulocytosis in patients with a human leukocyte antigen (HLA)-DQB1, which is a single-nucleotide polymorphism (i.e., 6672G > C) with high specificity and sensitivity rates [41] (Table 1). Another study proposed that the patients with a history of clozapine-induced granulocytopenia but without the variant HLA-B*59:01 may be successfully re-challenged with clozapine [255]. However, similar to results from the genetic studies investigating antipsychotic-induced TD, involvement of oxidative genes in bone marrow toxicity has also produced inconsistent results, as reflected by a marginal association with NADPH quinone 2 (*NQO2*) polymorphism [256] and negative results with myeloperoxidase [257,258]. An association with clozapine-induced agranulocytosis was also reported with tumor necrosis factor [259] but not with cytochrome b-245 α polypeptide [257] or with *CYPD26* [258] variants.

## 3. Pharmacogenomic (PGx) Studies

These studies have primarily explored the effects of genetically mediated PD differences in a drug’s response and/or adverse effects through a systematic assessment of genes, their products, and individual variation in gene expression and function. In this context, GWAS are most useful to generate hypotheses that can be later confirmed in future studies. These studies explore associations between multiple genes and psychotropic drugs [260]; therefore, they require large cohorts associated with high costs, which explains the scarcity of these studies with psychotropic medications. This may be the reason why most GWAS studies with antipsychotic drugs are primarily based on post hoc analyses from a large effectiveness trial, CATIE (Clinical Antipsychotic Trials of Intervention Effectiveness) [261,262,263,264,265]. Polymorphisms in the ankyrin repeat and sterile α motif domain containing 1B (*ANKS1B*) and contactin-associated protein-like 5 were linked to the efficacy of olanzapine and risperidone in terms of negative symptoms [265]. In addition, earlier GWAS identified genes from a region in chromosome 12 that were associated with antipsychotic-induced weight gain [266], GABAergic genes related to drug-induced TD [267], and HLA B*59:01 correlated with clozapine-induced agranulocytosis [255]. Another GWAS found 20 statistically significant polymorphisms at a single locus near the melanocortin 4 receptor (*MC4R*) gene associated with weight gain in patients undergoing the first trial with antipsychotic drugs, which is consistent with a region previously identified by large-scale GWAS of obesity in the general population [38]. These data implicate *MC4R* in extreme SGA-induced weight gain and related metabolic disturbances. Most recently, polygenic risk scores derived from significantly associated SNPs with schizophrenia patients of European ancestry were found to inversely correlate with the antipsychotic response [268]. It will be interesting to see if these results become more significant in specific sub-group analyses, such as males versus females, age at onset, and European versus non-European samples [268].

Antipsychotic treatment in a subset of 738 schizophrenia patients from the CATIE study [264] polymorphisms localized within or close to the genes, ETS homologous factor, solute carrier family 26 member 9 (*SLC26A9)*, DRD2, G protein-coupled receptor 137B, carbohydrate sulfotransferase 8, and interleukin1-alpha (IL1A) was associated with improvements in various neurocognitive domain areas. A significant result was also found for the variant rs286913 in the ETS homologous factor related to the effects of ziprasidone on vigilance. Furthermore, the presence of rs11240594 in the *SLC26A9* gene and rs11677416 in the *IL1A* gene was linked to olanzapine-induced improvement in processing speed and working memory.

## 4. Commercially Available Genetic Assays

These assays offer genetic testing for multiple genetic biomarkers (combinatorial assays) for treatment response and/or tolerability identified in other studies to facilitate the selection of effective psychotropic medications. The clinical utility of these combinatorial genetic assays has primarily been tested in underpowered and open-label studies. Thus, these assays with specific panels for different groups of psychotropic drugs are based on findings from other studies using a candidate gene(s) approach. Although there is no specific genetic assay for antipsychotic drugs, combinatorial genotyping of genetic biomarkers is used to optimize the efficacy and tolerability of antipsychotic drugs, especially in the treatment-refractory population. In this context, genetic variance in PK biomarkers (primarily the CYP enzyme system) has been clinically helpful to optimize antipsychotic treatment. Most genetically relevant CYP enzyme assays for antipsychotic drugs include CYP1A2, CYP2D6, and CYP2C19. *AmpliChip™* is the only FDA-approved genetic test, which is a microarray-based product to assess the activity of CYP2D6 and CYP2C19 and can be helpful in a large number of psychiatric patients as multiple psychotropic drugs are metabolized by these two CYP enzymes. Genetic testing for CYP2D6 is among the most clinically relevant investigation, as several important psychotropic drugs, including antipsychotic drugs, such as haloperidol, perphenazine, and risperidone, are metabolized by this enzyme. Following are the major resources and genetic assay companies that offer genetic testing for psychotropic drugs.

The GeneSight^®^ (Myriad Health^®^, South San Francisco, CA, USA) combinatorial assays provide coverage for about 50 PK alleles, including those for CYP2D6, CYP2C19, CYP2C9, CYP2B6, CYP3A4, and CYP1A2, and some PD genes (5HTT, HTR2A, COMT, CACNA1C, MTHFR). On the basis of information on these genetic biomarkers, an individualized report is created which divides psychotropic medications into a green bin for recommended use, a yellow bin for use with caution, and a red bin use with extreme caution and frequent monitoring.

Genecept^TM^ assay (Genomind^®^) also provides testing for PK biomarkers (CYP2D6, CYP2C19, CYP3A4) and PD markers, (5HT transporter, 5HT2C receptors, DRD2, COMT, CACNA1C, ANK3, and MTHFR). Like the GeneSight report, each patient’s results are provided to the ordering clinician, along with suggested therapeutic options.

Drug-Metabolizing Enzymes and Transporters (DMET™) Plus Solution is one of the largest commercially available genetic assays for about 2000 PK variants across multiple genes. The DMET™Plus Solution was developed as a platform to identify genetic variance and has not been tested for its efficacy in enhancing clinical outcomes with psychotropic drugs.

## 5. Future Directions

One of the most important goals for future genetic research in psychopharmacology will be to replicate and validate results from small sample genetic studies to resolve inconsistent results. However, these goals can only be accomplished by large, prospective, well-conducted multisite clinical trials such as genome-wide association studies to allow subgroup analyses and provide control for demographic, clinical, and environmental factors while close monitoring for medication adherence. In this context, the clinical trial network model used in oncology and cardiology has already been initiated in psychiatry, including Implementing Genomics in Practice (IGNITE), the Dutch Pharmacogenetics Working Group, and the Pharmacogenomic Resource for Enhanced Decisions in Care and Treatment. These large-scale initiatives will offer an effective tool to explore the relationship between efficacy and tolerability of various antipsychotic drugs and multiple genetic variants to generate hypotheses that could be tested in hypothesis-driven randomized controlled trials to improve and expand on currently available genetic biomarkers. A minimum set of genes and alleles is required for consistent pharmacogenomic decision-making before combinatorial genetic assays can be used beyond the treatment-refractory population. With the growing research in this area, it is only a matter of time that these efforts will help develop comprehensive combinatorial commercially available assays and toolkits with clinical utility beyond the treatment-refractory patient population facilitating the application of bench research to the clinical bedside. Equally importantly, clinicians must be trained and updated to enhance clinical application and interpretation of results from the existing and ongoing genetic studies with psychotropic medications, including antipsychotic drugs.

## Figures and Tables

**Table 1:** Genetic biomarkers for antipsychotic response and adverse effects.

Antipsychotic Response					
Gene	Polymorphism	Risk Allele	Functional Outcome	Clinical Outcome	Statistical Significance
DRD2	-141C Ins/Del (rs1799732)	Del	Decreased DRD2 expression	Lower antipsychotic response	Odds ratio = 0.65 95% confidence interval = 95% CI: 0.43–0.97 [26]
HTR1A	C-1019G	G	Increased HTR1A expression	G/G homozygosity with lesser negative symptom improvement [27,28,29,30]	*p* = 0.003
HTR2A	T-102-C (rs6313)	C	Decreased HTR2A expression	C/C homozygosity with lower antipsychotic response	Odds ratio = 0.61 95% confidence interval = 0.43–8.5 [31]
COMT	Val 158Met	Val	Faster metabolism resulting in lower levels of dopamine	Lower antipsychotic response [32]	Odds ratio = 1.37; 95% confidence interval = 1.02–1.85)
Weight Gain					
HTR2C	C-759T (rs3813929)	C	Lesser expression of HTR2C receptors [33]	>7% weight gain over baseline with C allele	Odds ratio = 1.64; 95% confidence interval = 0.73–3.69 in chronic subjects [34,35,36,37]; Odds ratio = 5.40 95% confidence interval = 2.08–14.01 during early psychosis [34,35,36,37].
MC4R	Rs489693	A	Unknown	AA homozygotes gained about 3 kg more weight than other genotypes [38]	Odds Ratio (95% confidence interval)
Tardive Dyskinesia					
CYP2D6	Presence of at least one dysfunctional alleles	One of 3, 4, 5, 6, or 10 alleles	Decreased CYP2D6 enzyme activity	Increased risk for tardive dyskinesia	1.83 95% CI: 1.09–3.08) [7,8,9,10,11,12,13,14,15,16,17,18,19,20,21]
HTR2A	T102C	C	Decreased HTR2A expression and binding	Presence of tardive dyskinesia	1.64 95% CI: 1.17–2.32 [39]
DRD2	Taq1A (rs1800497)	C, A2	Increased DRD2 receptors and binding	Presence of tardive dyskinesia	1.30 95% CI: 1.09–1.55 [40]
Agranulocytosis					
HLADQB1	G6672C (rs1133322494)	G	? autoimmune effect	Clozapine discontinuation due to ANC < 500 cells/mm^3^	Odds ratio = 16.9 [41]

## Abbreviations

Supplemental list for acronyms and abbreviations in alphabetical order

5-HTT LPR	serotonin-transporter-linked promoter region
CATIE	Clinical Antipsychotic Trials of Intervention Effectiveness
COMT	catechol-o-methyl transferase
CYP	cytochrome-P450
DAT	dopamine transporter
DRD1	dopamine-1 receptors
DRD2	dopamine-2 receptors
DRD3	dopamine-3 receptors
DRD4	dopamine-4 receptors
EHF	ETS homologous factor, solute carrier family 26 member 9 (*SLC26A9)*
EPS	extrapyramidal symptoms
GWAS	genome-wide association studies
HLA	human leukocyte antigen
HTR2A	serotonin-2A receptors
HTR2C	serotonin-2A receptors
IGNITE	Implementing Genomics in Practice
IL1A	interleukin1-alpha
MC4R	melanocortin 4 receptor
MDR1	multiple drug resistance-1
NADPH	nicotinamide adenine dinucleotide phosphate
PD	pharmacodynamic
PG	pharmacogenetic
PGx	pharmacogenomic
PK	pharmacokinetic
SLC26A9	solute carrier family 26 member 9
SLC6A4	solute carrier family 6 member 4—serotonin transporter gene
SNP	single nucleotide polymorphism
TD	tardive dyskinesia
